# Research on Gastroesophageal Reflux Disease Based on Dynamic Features of Ambulatory 24-Hour Esophageal pH Monitoring

**DOI:** 10.1155/2017/9239074

**Published:** 2017-11-14

**Authors:** Shuang Liu, Minpeng Xu, Jiajia Yang, Hongzhi Qi, Feng He, Xin Zhao, Peng Zhou, Lixin Zhang, Dong Ming

**Affiliations:** Neural Engineering & Rehabilitation Lab, Department of Biomedical Engineering, College of Precision Instruments and Optoelectronics Engineering, Tianjin University, Tianjin, China

## Abstract

Ambulatory 24-hour esophageal pH monitoring has been considered as the gold standard for diagnosing gastroesophageal reflux disease (GERD), and in clinical application, static parameters are widely used, such as DeMeester score. However, a shortcoming of these static variables is their relatively high false negative rate and long recording time required. They may be falsely labeled as nonrefluxers and not appropriately treated. Therefore, it is necessary to seek more accurate and objective parameters to detect and quantify GERD. This paper first describes a new effort that investigated the feasibility of dynamic features of 24-hour pH recording. Wavelet energy, information entropy, and wavelet entropy were estimated for three groups (severe, mild-to-moderate, and normal). The results suggest that wavelet energy and entropy are physiologically meaningful since they differentiated patients with varying degrees of GERD. *K*-means clustering algorithm was employed to obtain the sensitivity and specificity of new parameters. It is obvious that information entropy goes with the highest sensitivity of 87.3% and wavelet energy has the highest specificity of 97.1%. This would allow a more accurate definition of the best indicators to detect and quantify GERD as well as provide an alternative insight into the early diagnosis of GERD.

## 1. Introduction

Gastroesophageal reflux disease (GERD) [1] is an extremely common but complex disease characterized by recurrent reflux symptoms significantly associated with chest pain, dysphagia, dyspepsia, and globus sensation, leading to impaired quality of life, and is associated with a high disease burden [2]. GERD may increase the risk of developing bipolar disorder, sleep problems, and so on [3, 4]. It is reported that up to 7%–15% of people have the typical GERD symptoms of heartburn and regurgitation in western countries, with 2.5% to 7.1% in most population-based studies in Asia [5], notably about 5.77% in Beijing and Shanghai, China [6]. Furthermore, the incidence of reflux-induced esophageal cancer has increased more than eightfold in the past three decades [7]. Consequently, research on GERD has gradually attracted considerable attention over the past decade.

Current clinical diagnosis methods include 24-hour esophageal pH monitoring, gastroesophageal reflux scintigraphy, esophagus pressure test, and esophagus endoscopic test. Among these, 24-hour esophageal pH monitoring is currently the most reliable method for the detection and quantification of GERD for its relatively high sensitivity and specificity, even being considered as the gold standard for GERD diagnosis [8–11]. This test allows one to quantify the degree of gastroesophageal reflux in a near-physiological setting by measuring the frequency and duration of acid exposure to the esophageal mucosa and the length of time required to clear the esophagus of acid following a reflux episode. Ambulatory 24-hour pH monitoring could provide awfully important information about whether there exists pathological reflux, which plays a definitive role in the final diagnosis. Additionally, outpatient monitoring provides more physiological monitoring conditions, reduced costs, and simpler procedures compared with inpatient monitoring.

Many studies have confirmed the validity of esophageal pH in distinguishing GERD patients from normal subjects, but most of them focused on static parameters, such as DeMeester score and percentage of total monitoring time in which pH is below 4. The original scoring system devised by Johnson and DeMeester [12] examined six variables (number of reflux episodes, number of episodes longer than 5 minutes, longest reflux duration, total percentage of monitoring time with pH below 4, and the percentage of time with pH below 4 in an upright position and supine position, resp.) and calculated a composite score called the DeMeester score according to a formula dependent on the deviation of each of these six variables from normal values. Jamieson et al. [13] showed that the DeMeester score and the percentage of total time with pH < 4 provided the most efficient interpretation of the test. Robinson et al. [1] stressed the percentage of time with the pH below 4 as being the most useful outcome measure in discriminating physiological from pathological esophageal reflux, in line with some literatures [13, 14], but other variables which vary with the esophageal acid exposure time are less reproducible and have lower discriminatory power.

However, all these aforementioned variables have the shortcoming of relatively high false negative rates, including the percentage of time with pH < 4 and DeMeester score, and this is more of a problem in patients with nonerosive GERD [15]. So, they may be falsely labeled as nonrefluxers and not appropriately treated. The symptom index (SI) is another parameter used to evaluate the association of symptoms with acid reflux. SI is defined as the number of reflux-related symptom episodes divided by the total number of reflux episodes, expressed as a percentage. However, SI is not validated against an independent criterion of diagnostic accuracy such as symptomatic response to antireflux therapy [16]. Nonetheless, 24-hour esophageal pH monitoring is widely used for detection and quantification of GERD, but the abovementioned problems raise the question as to what the optimal parameters are to use for 24-hour pH monitoring to define and quantify GERD. In fact, the human body is a dynamic system and dynamic features of the internal environment may imply great physiological significance. Over the past few decades, static parameters have been intensively studied for 24-hour pH monitoring, but few concerned dynamic features which may yield the most accurate interpretation of the measurement.

In this paper, we describe a new effort that investigated the feasibility of dynamic features of pH recording obtained by wavelet transform and entropy theory. Wavelet energy, information entropy, and wavelet entropy were estimated for three groups—severe, mild-to-moderate, and normal—to explore optimal dynamic parameters for detecting and quantifying GERD. This would allow a more accurate definition of the best indicators to detect GERD as well as trying to discover a physiologically regular pattern. This may provide an alternative insight into the early diagnosis of GERD.

## 2. Materials and Methods 

### 2.1. Participants and Test

From 2009 to 2010, we evaluated 90 consecutive patients (50 males and 40 females) with suspected GERD who were first-time referrals to Nankai Hospital of Tianjin, aged 22 to 83 years (median age 53). These patients complaining of heartburn and regurgitation were primarily diagnosed with GERD, including outpatients and inpatients. None had scleroderma, esophageal achalasia, or diabetes mellitus or underwent gastrointestinal surgery, and selected patients were not allowed to take gastric motility stimulants or acid-inhibitory drugs for at least 48 hours prior to the study. The study was approved by the ethical committee of Tianjin University. All subjects signed informed consent in advance.

Ambulatory 24-hour esophageal pH monitoring was performed with a transnasally placed catheter to the distal esophageal mucosa. The pH of buffering solutions was used to calibrate the pH sensors at the beginning of the esophageal acid monitoring. The catheter type electrode was inserted through the nose and positioned 5 cm above the manometrically defined upper limit of the lower esophageal sphincter (LES) [16]. After placement of the pH electrode, the individuals were sent home and encouraged to perform normal daily activities. They were instructed to keep a diary for the next 24 hours that indicated the time of meals, when they assumed the supine position in preparation for sleep, when they arose in the morning, and when symptoms occurred; at the same time, subjects avoided consuming anything with pH < 4 in order to ensure standardization of the test. A consent form was completed by each subject before any procedure.

Furthermore, the data was recorded by a portable digital data logger for 24 hours with a sampling rate of 1/360 Hz while the patient was ambulatory, and it was analyzed for calculating the DeMeester score using a standard software program. An experienced expert evaluated the degree of acid reflux according to DeMeester score and the subject's symptoms. A score more than 14.72 was considered abnormal acid reflux, scores between 14.72 and 100 were regarded as mild-to-moderate GERD, and a score greater than 100 was regarded as severe GERD.

### 2.2. Data Processing

#### 2.2.1. DeMeester Score

DeMeester and Jonson determined the patients' scores based on the following six variables: number of reflux episodes, number of episodes longer than 5 minutes, maximal reflux duration, total percentage of time with pH below 4 for the total monitoring, and the percentage of time with pH below 4 in an upright position and supine position, respectively. The simplified formula for scoring each of the six variables is as follows:

(1) $$\begin{array}{c} \text{Scoring value}=\frac{X-A}{\text{SD}}+1. \end{array}$$

*X* is the detection value, *A* is the mean value, and SD is the standard deviation for each variable. This formula weighs each component of the 24 h record according to the dependability and reliability of the measurement. The composite score can be obtained by adding the scores calculated for each of the six components, called DeMeester score [17], where greater than 14.72 is considered abnormal, 14.72–50 is regarded as mild GERD, 51–100 is regarded as moderate GERD, and greater than 100 is regarded as severe GERD.

#### 2.2.2. Wavelet Transform

Wavelets can be literally defined as “small” waves that have limited duration and 0 average values. They are mathematical functions capable of localizing a function or a set of data in both time and frequency. The wavelet transformation is an effective tool in signal processing due to its attractive properties such as time and frequency localization and multirate filtering. Using these properties, one can extract the desired features from an input signal characterized by certain local properties in time and space [18].

A wavelet family *ψ*_*a*,*b*_(*t*) is the set of elementary functions generated by dilations and translations of a unique admissible mother wavelet *ψ*(*t*):

(2) $$\begin{array}{c} \psi_{a,b}\left(\;t\;\right)={\left|\;a\;\right|}^{-1/2}\psi \left(\;\frac{t-b}{a}\;\right). \end{array}$$

Then, the wavelet transformation of a signal *f*(*t*) is

(3) $$\begin{array}{c} W_{\psi }f\left(\;a,b\;\right)=\frac{1}{\sqrt{\left|a\right|}}\overset{\infty }{\underset{-\infty }{\int }}f\left(\;t\;\right)\psi^{*}\left(\;\frac{t-b}{a}\;\right)dt. \end{array}$$

In this work, Gaussian wavelet $\psi (t)=-(t/\sqrt{2\pi }\sigma^{3})exp({-x}^{2}/{2\sigma }^{2})$ was employed as the mother wavelet.

#### 2.2.3. Wavelet Energy

Since the family {*ψ*_*j*,*k*_(*t*)} is an orthonormal basis for *L*2 (*R*), the concept of energy is linked with the usual notions derived from Fourier theory. Then, wavelet coefficients are given by *Cj*(*k*) ={*S*, *ψ*_*j*,*k*_}, and the energy of a signal at each scale, *j* = −1,…, −*N*, will be

(4) $$\begin{array}{c} E_{j}={\left\|\;\gamma_{j}\;\right\|}^{2}=\underset{k}{\sum }{\left|\;C_{j}\left(\;k\;\right)\;\right|}^{2}. \end{array}$$

The energy at each sampled time *k* will be

(5) $$\begin{array}{c} E\left(\;k\;\right)=\overset{-1}{\underset{j=-N}{\sum }}{\left|\;C_{j}\left(\;k\;\right)\;\right|}^{2}. \end{array}$$

Consequently, the total energy can be obtained by

(6) $$\begin{array}{c} E_{\text{tol}}={\left\|\;S\;\right\|}^{2}=\underset{j<0}{\sum }\underset{k}{\sum }{\left|\;C_{j}\left(\;k\;\right)\;\right|}^{2}=\underset{j<0}{\sum }E_{j}. \end{array}$$

For the *j*th scale, the wavelet energy ratio is considered as a normalized value:

(7) $$\begin{array}{c} P_{j}=\frac{E_{j}}{E_{\text{tol}}}. \end{array}$$

The wavelet energy ratio vector {*p*_*j*_} represents energy distribution in a time scale, which gives a suitable tool for detecting and characterizing singular features of a signal in time-frequency domain [19]. Clearly, ∑_*j*=−1_^−*N*^*p*_*j*_ = 1.

#### 2.2.4. Information Entropy

The entropy can be interpreted not only as a measure of uncertainty but also as a measure of information. As a matter of fact, the amount of information which we get when we observe the result of an experiment (depending on chance) can be taken numerically equal to the amount of uncertainty concerning the outcome of the experiment before carrying it out. Shannon entropy, also called information entropy, gives a useful criterion for analyzing and comparing a probability distribution. It provides a measure of information of any distribution. It was calculated according to the following algorithm:

(8) $$\begin{array}{c} H=-\underset{i}{\sum }p_{i}\cdot \ln\left[\;p_{i}\;\right], \end{array}$$

where *i* extends over all observed amplitude values of the data time series and *p*_*i*_ is the probability that the amplitude value *v*_*i*_ occurs anywhere in the data time series. Therefore, *p*_*i*_ is the ratio of the number of data points with the amplitude value *v*_*i*_ to the total number of data points in the data time series [20].

#### 2.2.5. Wavelet Entropy

According to the Shannon entropy theory and wavelet energy ratio defined above, wavelet entropy is defined as

(9) $$\begin{array}{c} S_{WT}=S_{WT}\left(\;p\;\right)=-\underset{j<0}{\sum }p_{j}\cdot \ln\left[\;p_{j}\;\right]. \end{array}$$

To some extent, wavelet entropy can represent the degree of order/disorder associated with a multifrequency signal response, so it can provide useful information about the underlying dynamical process associated with measured signals [19]. For a general signal, a higher wavelet entropy suggests that wavelet energy of the measured signal is distributed more uniformly in frequency domain and vice versa.

#### 2.2.6. *K*-Means Clustering

Clustering is a method of unsupervised learning and a common technique for data analysis, and *K*-means is a well-known clustering algorithm for its simplicity and easy implementation [21]. *K*-means clustering algorithm [22, 23] aims at partitioning a given set of data into *k* disjoint subsets (clusters), such that the objects in the same cluster are as similar as possible and as dissimilar as possible in different clusters. Given a set of *n* data points in real *d*-dimensional space *R*^*d*^ and an integer* k*, the first step is to determine a set of* k* points in *R*^*d*^, called centers, so as to minimize the mean squared distance from each data point to its nearest center, where Euclidean distance is generally considered to determine the distance between data points and the centroids. And then the new centroids need to be recalculated as the inclusion of new points may lead to a change in the cluster centroids. The *k* centroids may change their position in a step-by-step manner until the centroids do not move anymore.

## 3. Results and Conclusion

### 3.1. Wave Patterns

**Figure 1:**
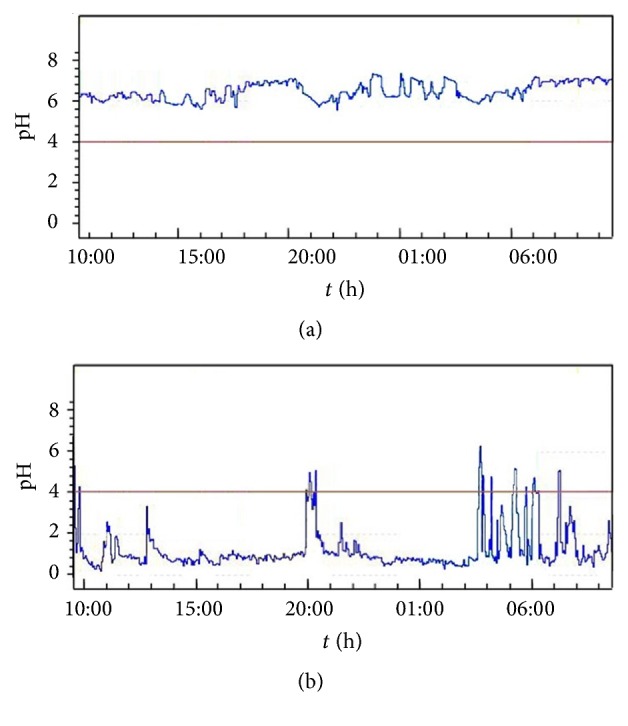
Measured pH signals from a normal subject (a) and a patient with pathological reflux (b) over the 24-hour monitoring period.

Figure 1 shows wave patterns of pH signals from one typical normal subject (top) and a patient with pathological reflux (bottom) over the total 24-hour monitoring period. It can be observed that normal individuals maintain the esophageal pH above 4 over the entire monitoring period, while for the patients, pH < 4 is a dominating trend with apparently significant fluctuations, but it should be noted that not all patients have the typical patterns.  Table 1 shows the sensitivity of the abovementioned static parameters. As can be seen, DeMeester scores and total percentage of monitoring time with pH below 4 get higher sensitivity among these parameters, 83.6% and 85.5%, respectively.

### 3.2. Dynamic Characteristics Analysis

**Figure 2:**
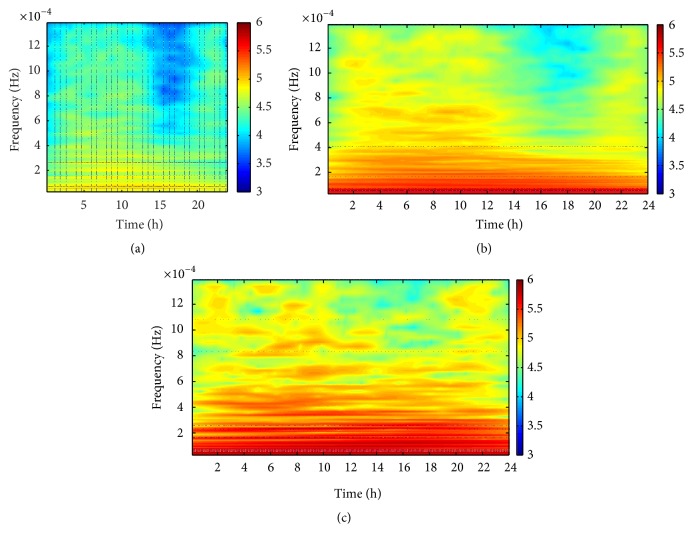
Group means of wavelet energy in time-frequency domain for (a) normal group, (b) mild-to-moderate group, and (c) severe group.

**Figure 3:**
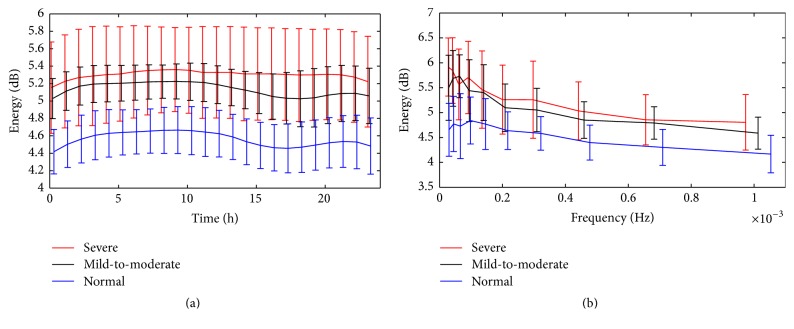
Group means of wavelet energy in (a) time domain and (b) frequency domain for each group.

Among the 90 subjects, there are 35 normal ones, 43 mild-to-moderate GERD patients, and 12 severe GERD patients. In order to detect frequency compositions of the pH signals and then identify the abnormalities, spectral analysis of the signals was performed using wavelet transformation in the time-frequency domain.  Figure 2 displays mean values of wavelet energy in the time-frequency domain for each group. Obviously, the energy is basically concentrated in the low frequency ranges whereas it is evenly distributed in the time domain for both the normal group and the patients, illustrated distinctly in the figure. Moreover, it can be observed that wavelet energy is significantly lower in the normal group than in the patient groups (*p* < 0.01). Additionally, wavelet energy was measured in time domain and frequency domain, respectively, as presented in Figure 3. Apparently, the severe group has the highest energy while the normal one has the lowest in both domains. From the above, we can ascertain that the energy of the pH signal increases accordingly with the deterioration of the GERD.

**Figure 4:**
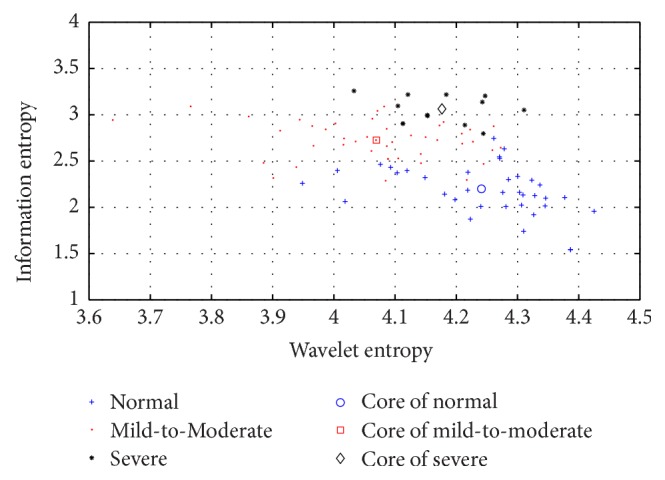
Values distribution of 90 individuals in the information entropy–wavelet entropy plane.

The information entropy and wavelet entropy of 90 individuals were then calculated.  Figure 4 shows the values distribution in the information entropy–wavelet entropy plane where three groups can be easily separated. As for information entropy, the severe group has the highest entropy while the normal group has the lowest, illustrating that pH signals of the patients with GERD are more irregular than that of normal subjects, especially for the severe group, whose pH signal fluctuates enormously, and thus they have the highest information entropy. However, wavelet entropy is highest in the normal group and lowest in the mild-to-moderate group, representing the notion that the energy distribution of pH recording in the frequency domain is relatively uniform for normal subjects, compared with the corresponding distribution associated with GERD. Furthermore, the present results demonstrate that wavelet energy and entropy are physiologically meaningful since they differentiated normal subjects from patients with differing degrees of GERD. An independent-samples *t*-test, following the normal distribution test, was performed. Significant statistical differences were found between every two groups (*p* < 0.05), and *p* values are listed in Table 2.

### 3.3. Performance of the Features

To quantitatively evaluate the performance of these dynamic features to identify GERD, *k*-means clustering algorithm was employed to obtain the sensitivity, specificity, and false negative rates of each feature, shown in Table 3. It is obvious that information entropy goes with the highest sensitivity of 87.3%, wavelet energy has the highest specificity of 97.1%, and information entropy has the lowest false negative rate of 12.7% accordingly. Wavelet entropy behaves the worst, having the lowest sensitivity and specificity, 58.2% and 80.0%, respectively. Compared with the DeMeester scores that got the highest sensitivity among the static parameters, information entropy got a relatively higher sensitivity. It should be noted that combining the three new parameters could improve the specificity, but this combination had the same sensitivity as the information entropy.

The correlation coefficients between these new features and DeMeester scores were calculated, 0.86% (*p* < 0.01), 0.841 (*p* < 0.01), and −0.413% (*p* < 0.01), respectively, for wavelet energy information entropy and wavelet entropy. That is, there was a positive correlation between DeMeester scores and the features of wavelet energy and information entropy and a negative correlation between DeMeester scores and wavelet entropy. Consequently, it can be mentioned that wavelet energy and information entropy have high performance for identifying GERD and they could be used in clinical and research areas.

## 4. Discussion

Pathological gastroesophageal reflux is a common disorder and the key to successful treatment is a reliable diagnosis. Long-term pH monitoring has been shown to be the most accurate test of gastroesophageal reflux disease [8–11] and ambulatory systems have proved to be safe and reliable according to several authors using different techniques [24–26].

The reported sensitivity of 24-hour esophageal pH monitoring in the literature varies significantly from 79% to 96% [8, 27–29], which may be related to daily activities and dietary restrictions. Several studies pointed out the high failure rate of the test to detect abnormal acid exposure in patients with endoscopically documented erosive esophagitis and suggested a lower sensitivity than previously reported (65%) [30, 31]. This high false negative rate is more of a problem in patients with nonerosive GERD. They may be falsely labeled as nonrefluxers and not appropriately treated. Therefore, it is necessary to seek more accurate and objective parameters to detect and quantify GERD.

In this work, we applied dynamic features to the study of GERD for the first time, including wavelet energy, information entropy, and wavelet entropy. New information has been accessed with approaches different from the traditional analysis of static parameters. Additionally, this work evaluated the sensitivity and specificity of the obtained characteristics for early GERD diagnosis. Acceptable results were got for wavelet energy and information entropy, 83.6% and 87.3% for sensitivity and 97.1% and 94.3% for specificity, respectively. Compared with the DeMeester scores, information entropy got a relatively higher sensitivity; moreover, it did not require the patients with suspected GERD to keep a diary for the next 24 hours that indicated the time of meals, when they assumed the supine position in preparation for sleep, when they arose in the morning, when symptoms occurred, and so on.

Still, 24-hour esophageal pH monitoring, despite suffering from some arguments such as a relatively high false negative rate, is currently the most commonly used method to monitor gastroesophageal reflux in many laboratories and hospitals around the world. The use of these dynamic features in our study offered an alternative insight into the early diagnosis of GERD. Further studies will develop 24-hour pH monitoring, with important implications for the evaluation of medical and surgical therapy.

## Figures and Tables

**Table 1:** Sensitivity of the parameters.

Variables	Sensitivity (%)
Total percentage of monitoring time with pH below 4 (%)	83.6
The percentage of time with pH below 4 in an upright position (%)	72.7
The percentage of time with pH below 4 in a supine position	65.5
Number of reflux episodes (times)	81.9
Number of episodes longer than 5 minutes (times)	74.5
Longest reflux duration (minutes)	69.1
DeMeester scores	85.5

**Table 2:** *p* values obtained from every two groups.

*p* values	Information entropy	Wavelet entropy
Normal versus mild-to-moderate	0.000	0.000
Normal versus severe	0.000	0.04
Mild-to-moderate versus severe	0.000	0.001

**Table 3:** Performance of the features.

	Wavelet energy	Information entropy	Wavelet entropy	All features
Sensitivity	83.6%	87.3%	58.2%	87.3%
Specificity	97.1%	94.3%	80.0%	100%
False negative rates	16.4%	12.7%	41.8%	12.7%
Correlation coefficient with DeMeester scores	0.860^*∗∗*^	0.841^*∗∗*^	−0.413^*∗∗*^	##

^*∗∗*^ *p* < 0.01; ^##^null.
